# Aqua­chloridobis(2-{[3-(morpholin-4-yl)prop­yl]imino­meth­yl}phenolato)manganese(III) monohydrate

**DOI:** 10.1107/S1600536811026493

**Published:** 2011-07-09

**Authors:** Nurul Azimah Ikmal Hisham, Hamid Khaledi, Hapipah Mohd Ali

**Affiliations:** a Department of Chemistry, University of Malaya, 50603 Kuala Lumpur, Malaysia

## Abstract

In the title compound, [Mn(C_14_H_19_N_2_O_2_)_2_Cl(H_2_O)]·H_2_O, the Mn^III^ atom is *N*,*O*-chelated by two monoanionic Schiff bases, forming two six-membered chelate rings. One Cl atom and one water mol­ecule in *trans* positions complete a distorted octa­hedral geometry around the metal atom. In the crystal, the complex mol­ecules and the uncoordinated water mol­ecules are connected *via* O—H⋯N, O—H⋯O and O—H⋯Cl hydrogen bonds into layers parallel to the *ac* plane and these are consolidated by C—H⋯π inter­actions. The layers are further linked into a three-dimensional network through C—H⋯O inter­actions.

## Related literature

For the structure of a Zn^II^ complex of the same Schiff base, see: Ikmal Hisham *et al.* (2011 ▶). For the structure of a similar Mn^III^ complex, see: Huang *et al.* (2004 ▶).
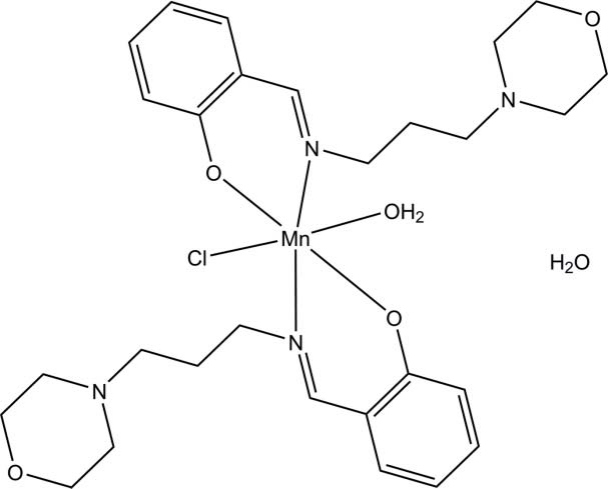


## Experimental

### 

#### Crystal data

[Mn(C_14_H_19_N_2_O_2_)_2_Cl(H_2_O)]·H_2_O
*M* *_r_* = 621.05
Triclinic, 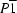

*a* = 9.4831 (2) Å
*b* = 12.4169 (3) Å
*c* = 12.9518 (3) Å
α = 95.540 (1)°
β = 90.306 (2)°
γ = 104.229 (1)°
*V* = 1470.72 (6) Å^3^
*Z* = 2
Mo *K*α radiation
μ = 0.59 mm^−1^
*T* = 100 K
0.20 × 0.16 × 0.04 mm

#### Data collection

Bruker APEXII CCD diffractometer
Absorption correction: multi-scan (*SADABS*; Sheldrick, 1996 ▶) *T*_min_ = 0.892, *T*_max_ = 0.977
13272 measured reflections
6393 independent reflections
4740 reflections with *I* > 2σ(*I*)
*R*_int_ = 0.034

#### Refinement

*R*[*F* ^2^ > 2σ(*F* ^2^)] = 0.039
*wR*(*F* ^2^) = 0.093
*S* = 0.99
6393 reflections
373 parameters
4 restraints
H atoms treated by a mixture of independent and constrained refinement
Δρ_max_ = 0.43 e Å^−3^
Δρ_min_ = −0.32 e Å^−3^

### 

Data collection: *APEX2* (Bruker, 2007 ▶); cell refinement: *SAINT* (Bruker, 2007 ▶); data reduction: *SAINT*; program(s) used to solve structure: *SHELXS97* (Sheldrick, 2008 ▶); program(s) used to refine structure: *SHELXL97* (Sheldrick, 2008 ▶); molecular graphics: *X-SEED* (Barbour, 2001 ▶); software used to prepare material for publication: *SHELXL97* and *publCIF* (Westrip, 2010 ▶).

## Supplementary Material

Crystal structure: contains datablock(s) I, global. DOI: 10.1107/S1600536811026493/is2745sup1.cif

Structure factors: contains datablock(s) I. DOI: 10.1107/S1600536811026493/is2745Isup2.hkl

Additional supplementary materials:  crystallographic information; 3D view; checkCIF report

## Figures and Tables

**Table 1:** Hydrogen-bond geometry (Å, °) *Cg*1 is the centroid of the C15–C20 ring.

*D*—H⋯*A*	*D*—H	H⋯*A*	*D*⋯*A*	*D*—H⋯*A*
O5—H5*A*⋯O6	0.83 (2)	1.88 (2)	2.709 (2)	174 (3)
O5—H5*B*⋯N2^i^	0.82 (2)	2.08 (2)	2.886 (2)	170 (2)
O6—H6*A*⋯Cl1^ii^	0.84 (2)	2.34 (2)	3.1761 (16)	178 (2)
O6—H6*B*⋯N4^iii^	0.86 (2)	1.99 (2)	2.834 (2)	169 (2)
C8—H8*B*⋯O6	0.99	2.56	3.551 (3)	174
C22—H22*B*⋯O5	0.99	2.51	3.154 (3)	123
C3—H3⋯O4^iv^	0.95	2.46	3.171 (3)	132
C9—H9*A*⋯O6^i^	0.99	2.58	3.471 (3)	150
C27—H27*B*⋯O2^v^	0.99	2.55	3.488 (3)	159
C23—H23*B*⋯*Cg*1^vi^	0.99	2.94	3.764	141

Symmetry codes: (i) 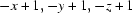; (ii) 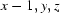; (iii) 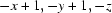; (iv) 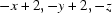; (v) 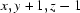; (vi) 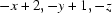.

**Fig. 1.**
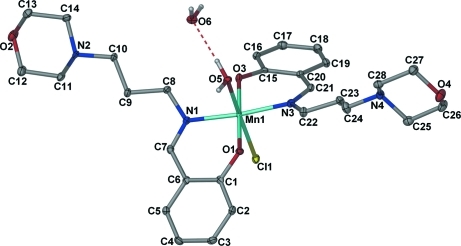
The molecular structure of the title compound with displacement ellipsoids drawn at 50° probability level. The C-bound Hydrogen atoms have been omitted for clarity.

## supplementary crystallographic information

### Comment

The title Mn^III^ complex was obtained through the reaction of the Schiff base, prepared *in situ*, and Mn(II) chloride. Under the reaction conditions, Mn^II^ ion was oxidized to Mn^III^ and *N,O*-chelated by two deprotonated Schiff base ligands. Similar to what was observed in a Zn(II) complex of the same Schiff base (Ikmal Hisham *et al.*, 2011), the ligand applies only its phenolate oxygen and imine nitrogen atoms in the coordination, while its morpholine nitrogen atom stays away from coordination. One chlorine atom and one molecule of water complete the distorted octahedral coordination environment. The Mn—N, Mn—O and Mn—Cl interatomic distances are comparable to the values reported for a similar structure (Huang *et al.*, 2004). In the crystal, the Mn^III^ complexes and the hydration water molecules are hydrogen bonded together through O—H···N, O—H···O and O—H···Cl interactions, forming two-dimensional arrays parallel to the *ac* plane. The structure of the layers is supplemented by C—H···π interactions (Table 1). The layers are further linked into a three-dimensional network *via* C—H···O interactions.

### Experimental

A mixture of salicylaldehyde (0.20 g, 1.64 mmol) and *N*-(3-aminopropyl)morpholine (0.24 g, 1.64 mmol) in ethanol (20 ml) was refluxed for 2 hr followed by addition of a solution of manganese(II) chloride (0.21 g, 1.64 mmol) in a minimum amount of water. The resulting solution was stirred for 2 hr at room temperature and then set aside. The crystals of the title complex were obtained after one week

### Refinement

The C-bound hydrogen atoms were placed at calculated positions and refined as riding atoms, with C—H distances of 0.95 (aryl) and 0.99 (methylene) Å. The O-bound hydrogen atoms were located in a difference Fourier map and refined, with a distance restraint of O—H = 0.84 (2) Å. For all hydrogen atoms *U*_iso_(H) were set to 1.2 (1.5 for hydroxyl)*U*_eq_(carrier atoms). The most disagreeable reflections with delta(F2)/e.s.d. > 10 were omitted (3 reflections).

### Crystal data

**Table:**

[Mn(C_14_H_19_N_2_O_2_)_2_Cl(H_2_O)]·H_2_O	*Z* = 2
*M**_r_* = 621.05	*F*(000) = 656
Triclinic, *P*1	*D*_x_ = 1.402 Mg m^−^^3^
Hall symbol: -P 1	Mo *K*α radiation, λ = 0.71073 Å
*a* = 9.4831 (2) Å	Cell parameters from 3220 reflections
*b* = 12.4169 (3) Å	θ = 2.2–28.9°
*c* = 12.9518 (3) Å	µ = 0.59 mm^−^^1^
α = 95.540 (1)°	*T* = 100 K
β = 90.306 (2)°	Plate, blue
γ = 104.229 (1)°	0.20 × 0.16 × 0.04 mm
*V* = 1470.72 (6) Å^3^	

### Data collection

**Table:**

Bruker APEXII CCD diffractometer	6393 independent reflections
Radiation source: fine-focus sealed tube	4740 reflections with *I* > 2σ(*I*)
graphite	*R*_int_ = 0.034
φ and ω scans	θ_max_ = 27.0°, θ_min_ = 2.2°
Absorption correction: multi-scan (*SADABS*; Sheldrick, 1996)	*h* = −12→12
*T*_min_ = 0.892, *T*_max_ = 0.977	*k* = −15→15
13272 measured reflections	*l* = −16→16

### Refinement

**Table:**

Refinement on *F*^2^	Primary atom site location: structure-invariant direct methods
Least-squares matrix: full	Secondary atom site location: difference Fourier map
*R*[*F*^2^ > 2σ(*F*^2^)] = 0.039	Hydrogen site location: inferred from neighbouring sites
*wR*(*F*^2^) = 0.093	H atoms treated by a mixture of independent and constrained refinement
*S* = 0.99	*w* = 1/[σ^2^(*F*_o_^2^) + (0.0463*P*)^2^] where *P* = (*F*_o_^2^ + 2*F*_c_^2^)/3
6393 reflections	(Δ/σ)_max_ = 0.001
373 parameters	Δρ_max_ = 0.43 e Å^−^^3^
4 restraints	Δρ_min_ = −0.32 e Å^−^^3^

### Special details

**Table:**

Geometry. All e.s.d.'s (except the e.s.d. in the dihedral angle between two l.s. planes) are estimated using the full covariance matrix. The cell e.s.d.'s are taken into account individually in the estimation of e.s.d.'s in distances, angles and torsion angles; correlations between e.s.d.'s in cell parameters are only used when they are defined by crystal symmetry. An approximate (isotropic) treatment of cell e.s.d.'s is used for estimating e.s.d.'s involving l.s. planes.
Refinement. Refinement of *F*^2^ against ALL reflections. The weighted *R*-factor *wR* and goodness of fit *S* are based on *F*^2^, conventional *R*-factors *R* are based on *F*, with *F* set to zero for negative *F*^2^. The threshold expression of *F*^2^ > σ(*F*^2^) is used only for calculating *R*-factors(gt) *etc*. and is not relevant to the choice of reflections for refinement. *R*-factors based on *F*^2^ are statistically about twice as large as those based on *F*, and *R*- factors based on ALL data will be even larger.

### Fractional atomic coordinates and isotropic or equivalent isotropic displacement parameters (Å^2^)

**Table:**

	*x*	*y*	*z*	*U*_iso_*/*U*_eq_	
Mn1	0.80758 (3)	0.51790 (3)	0.25109 (2)	0.00991 (9)	
Cl1	1.07112 (5)	0.49604 (5)	0.24934 (4)	0.01422 (12)	
O1	0.88326 (15)	0.67047 (12)	0.29100 (11)	0.0120 (3)	
O2	0.37546 (17)	0.08392 (13)	0.79286 (12)	0.0206 (4)	
O3	0.73429 (16)	0.36491 (12)	0.21182 (11)	0.0132 (3)	
O4	0.71302 (17)	0.94441 (14)	−0.31588 (12)	0.0229 (4)	
O5	0.58953 (16)	0.55756 (13)	0.24999 (12)	0.0136 (3)	
H5A	0.511 (2)	0.5122 (18)	0.2587 (19)	0.020*	
H5B	0.585 (3)	0.6146 (16)	0.2859 (17)	0.020*	
N1	0.77786 (18)	0.49029 (14)	0.40365 (13)	0.0107 (4)	
N2	0.45557 (18)	0.24065 (15)	0.64027 (13)	0.0118 (4)	
N3	0.81100 (18)	0.54400 (15)	0.09684 (13)	0.0118 (4)	
N4	0.71767 (19)	0.80497 (15)	−0.15371 (14)	0.0136 (4)	
C1	0.9646 (2)	0.71444 (18)	0.37568 (16)	0.0112 (4)	
C2	1.0615 (2)	0.81922 (18)	0.37504 (16)	0.0139 (5)	
H2	1.0708	0.8558	0.3134	0.017*	
C3	1.1439 (2)	0.87054 (19)	0.46231 (17)	0.0153 (5)	
H3	1.2097	0.9415	0.4597	0.018*	
C4	1.1319 (2)	0.81970 (19)	0.55433 (17)	0.0155 (5)	
H4	1.1888	0.8555	0.6142	0.019*	
C5	1.0359 (2)	0.71650 (18)	0.55720 (16)	0.0134 (4)	
H5	1.0261	0.6819	0.6199	0.016*	
C6	0.9528 (2)	0.66207 (18)	0.46865 (16)	0.0113 (4)	
C7	0.8498 (2)	0.55633 (18)	0.47924 (16)	0.0121 (4)	
H7	0.8341	0.5340	0.5472	0.015*	
C8	0.6650 (2)	0.39039 (18)	0.42446 (16)	0.0130 (4)	
H8A	0.6932	0.3242	0.3906	0.016*	
H8B	0.5731	0.3941	0.3900	0.016*	
C9	0.6333 (2)	0.37064 (18)	0.53695 (16)	0.0137 (5)	
H9A	0.6052	0.4355	0.5737	0.016*	
H9B	0.7209	0.3601	0.5726	0.016*	
C10	0.5093 (2)	0.26629 (18)	0.53680 (16)	0.0138 (5)	
H10A	0.4274	0.2753	0.4935	0.017*	
H10B	0.5425	0.2018	0.5039	0.017*	
C11	0.5658 (2)	0.20893 (19)	0.70355 (17)	0.0155 (5)	
H11A	0.6521	0.2728	0.7163	0.019*	
H11B	0.5971	0.1460	0.6659	0.019*	
C12	0.5019 (2)	0.1749 (2)	0.80625 (17)	0.0192 (5)	
H12A	0.5764	0.1534	0.8481	0.023*	
H12B	0.4762	0.2397	0.8451	0.023*	
C13	0.2678 (2)	0.11214 (19)	0.73002 (17)	0.0164 (5)	
H13A	0.2352	0.1750	0.7668	0.020*	
H13B	0.1824	0.0474	0.7187	0.020*	
C14	0.3271 (2)	0.14476 (19)	0.62656 (17)	0.0152 (5)	
H14A	0.3544	0.0805	0.5879	0.018*	
H14B	0.2507	0.1645	0.5852	0.018*	
C15	0.7750 (2)	0.30723 (18)	0.13036 (16)	0.0114 (4)	
C16	0.7705 (2)	0.19455 (18)	0.13548 (16)	0.0133 (5)	
H16	0.7444	0.1618	0.1982	0.016*	
C17	0.8037 (2)	0.13013 (19)	0.05026 (17)	0.0169 (5)	
H17	0.8005	0.0538	0.0553	0.020*	
C18	0.8416 (2)	0.17592 (19)	−0.04295 (17)	0.0169 (5)	
H18	0.8638	0.1311	−0.1013	0.020*	
C19	0.8466 (2)	0.28655 (19)	−0.04938 (16)	0.0149 (5)	
H19	0.8713	0.3177	−0.1130	0.018*	
C20	0.8160 (2)	0.35454 (18)	0.03621 (16)	0.0119 (4)	
C21	0.8181 (2)	0.46903 (18)	0.02342 (16)	0.0127 (4)	
H21	0.8255	0.4905	−0.0452	0.015*	
C22	0.7958 (2)	0.65460 (18)	0.07310 (16)	0.0146 (5)	
H22A	0.8813	0.7115	0.1043	0.018*	
H22B	0.7088	0.6688	0.1084	0.018*	
C23	0.7820 (2)	0.67320 (18)	−0.03982 (16)	0.0152 (5)	
H23A	0.7043	0.6123	−0.0753	0.018*	
H23B	0.8747	0.6728	−0.0746	0.018*	
C24	0.7455 (3)	0.78488 (19)	−0.04681 (17)	0.0189 (5)	
H24A	0.6583	0.7872	−0.0057	0.023*	
H24B	0.8273	0.8455	−0.0159	0.023*	
C25	0.8518 (2)	0.85933 (19)	−0.20428 (17)	0.0179 (5)	
H25A	0.9215	0.8115	−0.2056	0.022*	
H25B	0.8980	0.9317	−0.1644	0.022*	
C26	0.8160 (2)	0.8783 (2)	−0.31383 (18)	0.0206 (5)	
H26A	0.9063	0.9162	−0.3467	0.025*	
H26B	0.7762	0.8053	−0.3546	0.025*	
C27	0.5831 (2)	0.8915 (2)	−0.26777 (17)	0.0184 (5)	
H27A	0.5393	0.8184	−0.3071	0.022*	
H27B	0.5122	0.9382	−0.2693	0.022*	
C28	0.6134 (2)	0.87446 (18)	−0.15698 (17)	0.0149 (5)	
H28A	0.6536	0.9477	−0.1167	0.018*	
H28B	0.5216	0.8377	−0.1252	0.018*	
O6	0.34610 (17)	0.39900 (13)	0.28380 (12)	0.0158 (3)	
H6A	0.274 (2)	0.426 (2)	0.2761 (19)	0.024*	
H6B	0.339 (3)	0.3387 (16)	0.2441 (17)	0.024*	

### Atomic displacement parameters (Å^2^)

**Table:**

	*U*^11^	*U*^22^	*U*^33^	*U*^12^	*U*^13^	*U*^23^
Mn1	0.01027 (17)	0.01079 (18)	0.00858 (17)	0.00230 (13)	0.00173 (12)	0.00125 (12)
Cl1	0.0121 (2)	0.0199 (3)	0.0121 (3)	0.0063 (2)	0.00197 (19)	0.0027 (2)
O1	0.0125 (7)	0.0103 (8)	0.0118 (8)	0.0000 (6)	−0.0014 (6)	0.0014 (6)
O2	0.0196 (8)	0.0183 (9)	0.0260 (9)	0.0054 (7)	0.0063 (7)	0.0107 (7)
O3	0.0172 (8)	0.0118 (8)	0.0102 (8)	0.0025 (7)	0.0046 (6)	0.0014 (6)
O4	0.0211 (9)	0.0252 (10)	0.0267 (10)	0.0090 (8)	0.0071 (7)	0.0144 (7)
O5	0.0113 (7)	0.0135 (9)	0.0149 (8)	0.0017 (7)	0.0026 (6)	−0.0005 (6)
N1	0.0104 (9)	0.0103 (9)	0.0123 (9)	0.0034 (7)	0.0025 (7)	0.0028 (7)
N2	0.0099 (9)	0.0129 (9)	0.0114 (9)	−0.0004 (7)	0.0034 (7)	0.0026 (7)
N3	0.0104 (9)	0.0125 (9)	0.0130 (9)	0.0039 (8)	0.0022 (7)	0.0015 (7)
N4	0.0144 (9)	0.0146 (10)	0.0132 (9)	0.0055 (8)	0.0018 (7)	0.0034 (7)
C1	0.0093 (10)	0.0147 (11)	0.0112 (11)	0.0065 (9)	0.0019 (8)	−0.0009 (8)
C2	0.0146 (11)	0.0143 (12)	0.0127 (11)	0.0029 (9)	0.0035 (9)	0.0038 (9)
C3	0.0116 (10)	0.0130 (11)	0.0195 (12)	0.0004 (9)	0.0017 (9)	−0.0006 (9)
C4	0.0154 (11)	0.0171 (12)	0.0126 (11)	0.0030 (10)	−0.0021 (9)	−0.0029 (9)
C5	0.0145 (11)	0.0143 (11)	0.0114 (11)	0.0038 (9)	0.0017 (8)	0.0018 (9)
C6	0.0092 (10)	0.0144 (11)	0.0112 (11)	0.0051 (9)	0.0026 (8)	0.0008 (8)
C7	0.0122 (10)	0.0150 (11)	0.0108 (11)	0.0060 (9)	0.0022 (8)	0.0029 (9)
C8	0.0116 (10)	0.0145 (11)	0.0120 (11)	0.0009 (9)	0.0003 (8)	0.0028 (9)
C9	0.0141 (11)	0.0149 (12)	0.0118 (11)	0.0024 (9)	0.0033 (8)	0.0029 (9)
C10	0.0140 (11)	0.0149 (12)	0.0110 (11)	0.0004 (9)	0.0031 (8)	0.0026 (9)
C11	0.0106 (10)	0.0199 (12)	0.0165 (12)	0.0033 (9)	0.0031 (8)	0.0062 (9)
C12	0.0150 (11)	0.0259 (13)	0.0183 (12)	0.0054 (10)	0.0016 (9)	0.0081 (10)
C13	0.0141 (11)	0.0149 (12)	0.0204 (12)	0.0038 (9)	0.0050 (9)	0.0023 (9)
C14	0.0131 (11)	0.0142 (11)	0.0161 (12)	0.0001 (9)	0.0023 (9)	−0.0013 (9)
C15	0.0073 (10)	0.0134 (11)	0.0116 (11)	0.0005 (9)	−0.0015 (8)	−0.0026 (8)
C16	0.0119 (10)	0.0154 (12)	0.0121 (11)	0.0017 (9)	0.0006 (8)	0.0031 (9)
C17	0.0159 (11)	0.0145 (12)	0.0198 (12)	0.0036 (9)	−0.0007 (9)	−0.0005 (9)
C18	0.0185 (11)	0.0166 (12)	0.0149 (12)	0.0048 (10)	0.0021 (9)	−0.0027 (9)
C19	0.0156 (11)	0.0177 (12)	0.0108 (11)	0.0034 (10)	0.0009 (8)	0.0001 (9)
C20	0.0107 (10)	0.0127 (11)	0.0117 (11)	0.0018 (9)	0.0002 (8)	0.0014 (8)
C21	0.0107 (10)	0.0164 (12)	0.0105 (11)	0.0021 (9)	0.0018 (8)	0.0026 (9)
C22	0.0178 (11)	0.0146 (12)	0.0129 (11)	0.0061 (10)	0.0031 (9)	0.0031 (9)
C23	0.0180 (11)	0.0165 (12)	0.0117 (11)	0.0054 (10)	−0.0017 (9)	0.0007 (9)
C24	0.0258 (13)	0.0193 (13)	0.0137 (12)	0.0093 (11)	−0.0006 (9)	0.0026 (9)
C25	0.0153 (11)	0.0167 (12)	0.0221 (13)	0.0049 (10)	0.0012 (9)	0.0013 (10)
C26	0.0180 (12)	0.0233 (13)	0.0230 (13)	0.0062 (10)	0.0062 (10)	0.0106 (10)
C27	0.0168 (11)	0.0202 (13)	0.0191 (12)	0.0051 (10)	0.0010 (9)	0.0051 (10)
C28	0.0131 (11)	0.0127 (11)	0.0186 (12)	0.0031 (9)	0.0001 (9)	0.0008 (9)
O6	0.0141 (8)	0.0135 (9)	0.0199 (9)	0.0046 (7)	−0.0012 (7)	−0.0014 (7)

### Geometric parameters (Å, °)

**Table:**

Mn1—O1	1.8735 (15)	C10—H10B	0.9900
Mn1—O3	1.8738 (15)	C11—C12	1.519 (3)
Mn1—N1	2.0471 (17)	C11—H11A	0.9900
Mn1—N3	2.0537 (17)	C11—H11B	0.9900
Mn1—O5	2.2406 (15)	C12—H12A	0.9900
Mn1—Cl1	2.5793 (6)	C12—H12B	0.9900
O1—C1	1.328 (2)	C13—C14	1.509 (3)
O2—C12	1.428 (3)	C13—H13A	0.9900
O2—C13	1.431 (3)	C13—H13B	0.9900
O3—C15	1.330 (2)	C14—H14A	0.9900
O4—C26	1.423 (3)	C14—H14B	0.9900
O4—C27	1.424 (3)	C15—C16	1.397 (3)
O5—H5A	0.829 (16)	C15—C20	1.419 (3)
O5—H5B	0.819 (16)	C16—C17	1.383 (3)
N1—C7	1.288 (3)	C16—H16	0.9500
N1—C8	1.474 (3)	C17—C18	1.395 (3)
N2—C10	1.472 (2)	C17—H17	0.9500
N2—C11	1.475 (3)	C18—C19	1.373 (3)
N2—C14	1.476 (3)	C18—H18	0.9500
N3—C21	1.279 (3)	C19—C20	1.403 (3)
N3—C22	1.476 (3)	C19—H19	0.9500
N4—C24	1.464 (3)	C20—C21	1.442 (3)
N4—C28	1.466 (3)	C21—H21	0.9500
N4—C25	1.475 (3)	C22—C23	1.512 (3)
C1—C2	1.397 (3)	C22—H22A	0.9900
C1—C6	1.415 (3)	C22—H22B	0.9900
C2—C3	1.379 (3)	C23—C24	1.520 (3)
C2—H2	0.9500	C23—H23A	0.9900
C3—C4	1.394 (3)	C23—H23B	0.9900
C3—H3	0.9500	C24—H24A	0.9900
C4—C5	1.381 (3)	C24—H24B	0.9900
C4—H4	0.9500	C25—C26	1.511 (3)
C5—C6	1.404 (3)	C25—H25A	0.9900
C5—H5	0.9500	C25—H25B	0.9900
C6—C7	1.450 (3)	C26—H26A	0.9900
C7—H7	0.9500	C26—H26B	0.9900
C8—C9	1.520 (3)	C27—C28	1.507 (3)
C8—H8A	0.9900	C27—H27A	0.9900
C8—H8B	0.9900	C27—H27B	0.9900
C9—C10	1.521 (3)	C28—H28A	0.9900
C9—H9A	0.9900	C28—H28B	0.9900
C9—H9B	0.9900	O6—H6A	0.840 (16)
C10—H10A	0.9900	O6—H6B	0.855 (16)
			
O1—Mn1—O3	179.21 (7)	C11—C12—H12A	109.1
O1—Mn1—N1	89.85 (7)	O2—C12—H12B	109.1
O3—Mn1—N1	89.94 (7)	C11—C12—H12B	109.1
O1—Mn1—N3	91.65 (7)	H12A—C12—H12B	107.9
O3—Mn1—N3	88.65 (7)	O2—C13—C14	111.31 (17)
N1—Mn1—N3	173.12 (7)	O2—C13—H13A	109.4
O1—Mn1—O5	85.88 (6)	C14—C13—H13A	109.4
O3—Mn1—O5	94.87 (6)	O2—C13—H13B	109.4
N1—Mn1—O5	87.84 (6)	C14—C13—H13B	109.4
N3—Mn1—O5	85.57 (6)	H13A—C13—H13B	108.0
O1—Mn1—Cl1	88.07 (5)	N2—C14—C13	111.01 (18)
O3—Mn1—Cl1	91.19 (5)	N2—C14—H14A	109.4
N1—Mn1—Cl1	94.78 (5)	C13—C14—H14A	109.4
N3—Mn1—Cl1	91.99 (5)	N2—C14—H14B	109.4
O5—Mn1—Cl1	173.41 (4)	C13—C14—H14B	109.4
C1—O1—Mn1	126.07 (13)	H14A—C14—H14B	108.0
C12—O2—C13	110.02 (16)	O3—C15—C16	119.06 (18)
C15—O3—Mn1	125.73 (13)	O3—C15—C20	122.30 (19)
C26—O4—C27	109.95 (17)	C16—C15—C20	118.56 (18)
Mn1—O5—H5A	124.5 (17)	C17—C16—C15	120.7 (2)
Mn1—O5—H5B	115.0 (18)	C17—C16—H16	119.6
H5A—O5—H5B	105 (2)	C15—C16—H16	119.6
C7—N1—C8	120.36 (18)	C16—C17—C18	120.8 (2)
C7—N1—Mn1	123.24 (15)	C16—C17—H17	119.6
C8—N1—Mn1	116.38 (13)	C18—C17—H17	119.6
C10—N2—C11	111.46 (16)	C19—C18—C17	119.2 (2)
C10—N2—C14	108.09 (16)	C19—C18—H18	120.4
C11—N2—C14	107.89 (17)	C17—C18—H18	120.4
C21—N3—C22	120.34 (18)	C18—C19—C20	121.3 (2)
C21—N3—Mn1	123.59 (15)	C18—C19—H19	119.4
C22—N3—Mn1	115.98 (13)	C20—C19—H19	119.4
C24—N4—C28	110.73 (16)	C19—C20—C15	119.3 (2)
C24—N4—C25	112.32 (17)	C19—C20—C21	118.59 (19)
C28—N4—C25	108.87 (17)	C15—C20—C21	121.96 (18)
O1—C1—C2	118.93 (19)	N3—C21—C20	125.36 (19)
O1—C1—C6	122.81 (19)	N3—C21—H21	117.3
C2—C1—C6	118.19 (19)	C20—C21—H21	117.3
C3—C2—C1	121.2 (2)	N3—C22—C23	117.68 (17)
C3—C2—H2	119.4	N3—C22—H22A	107.9
C1—C2—H2	119.4	C23—C22—H22A	107.9
C2—C3—C4	120.9 (2)	N3—C22—H22B	107.9
C2—C3—H3	119.6	C23—C22—H22B	107.9
C4—C3—H3	119.6	H22A—C22—H22B	107.2
C5—C4—C3	119.1 (2)	C22—C23—C24	109.22 (17)
C5—C4—H4	120.5	C22—C23—H23A	109.8
C3—C4—H4	120.5	C24—C23—H23A	109.8
C4—C5—C6	120.9 (2)	C22—C23—H23B	109.8
C4—C5—H5	119.5	C24—C23—H23B	109.8
C6—C5—H5	119.5	H23A—C23—H23B	108.3
C5—C6—C1	119.8 (2)	N4—C24—C23	112.68 (17)
C5—C6—C7	117.55 (19)	N4—C24—H24A	109.1
C1—C6—C7	122.54 (19)	C23—C24—H24A	109.1
N1—C7—C6	125.10 (19)	N4—C24—H24B	109.1
N1—C7—H7	117.5	C23—C24—H24B	109.1
C6—C7—H7	117.5	H24A—C24—H24B	107.8
N1—C8—C9	117.99 (18)	N4—C25—C26	109.81 (18)
N1—C8—H8A	107.8	N4—C25—H25A	109.7
C9—C8—H8A	107.8	C26—C25—H25A	109.7
N1—C8—H8B	107.8	N4—C25—H25B	109.7
C9—C8—H8B	107.8	C26—C25—H25B	109.7
H8A—C8—H8B	107.1	H25A—C25—H25B	108.2
C8—C9—C10	107.43 (17)	O4—C26—C25	111.65 (18)
C8—C9—H9A	110.2	O4—C26—H26A	109.3
C10—C9—H9A	110.2	C25—C26—H26A	109.3
C8—C9—H9B	110.2	O4—C26—H26B	109.3
C10—C9—H9B	110.2	C25—C26—H26B	109.3
H9A—C9—H9B	108.5	H26A—C26—H26B	108.0
N2—C10—C9	114.57 (17)	O4—C27—C28	111.13 (18)
N2—C10—H10A	108.6	O4—C27—H27A	109.4
C9—C10—H10A	108.6	C28—C27—H27A	109.4
N2—C10—H10B	108.6	O4—C27—H27B	109.4
C9—C10—H10B	108.6	C28—C27—H27B	109.4
H10A—C10—H10B	107.6	H27A—C27—H27B	108.0
N2—C11—C12	109.64 (17)	N4—C28—C27	109.93 (17)
N2—C11—H11A	109.7	N4—C28—H28A	109.7
C12—C11—H11A	109.7	C27—C28—H28A	109.7
N2—C11—H11B	109.7	N4—C28—H28B	109.7
C12—C11—H11B	109.7	C27—C28—H28B	109.7
H11A—C11—H11B	108.2	H28A—C28—H28B	108.2
O2—C12—C11	112.40 (19)	H6A—O6—H6B	111 (2)
O2—C12—H12A	109.1		

### Hydrogen-bond geometry (Å, °)

**Table:**

*Cg*1 is the centroid of the C15–C20 ring.

**Table:**

*D*—H···*A*	*D*—H	H···*A*	*D*···*A*	*D*—H···*A*
O5—H5A···O6	0.83 (2)	1.88 (2)	2.709 (2)	174 (3)
O5—H5B···N2^i^	0.82 (2)	2.08 (2)	2.886 (2)	170 (2)
O6—H6A···Cl1^ii^	0.84 (2)	2.34 (2)	3.1761 (16)	178 (2)
O6—H6B···N4^iii^	0.86 (2)	1.99 (2)	2.834 (2)	169 (2)
C8—H8B···O6	0.99	2.56	3.551 (3)	174
C22—H22B···O5	0.99	2.51	3.154 (3)	123
C3—H3···O4^iv^	0.95	2.46	3.171 (3)	132
C9—H9A···O6^i^	0.99	2.58	3.471 (3)	150
C27—H27B···O2^v^	0.99	2.55	3.488 (3)	159
C23—H23B···Cg1^vi^	0.99	2.94	3.764	141

Symmetry codes: (i) −*x*+1, −*y*+1, −*z*+1; (ii) *x*−1, *y*, *z*; (iii) −*x*+1, −*y*+1, −*z*; (iv) −*x*+2, −*y*+2, −*z*; (v) *x*, *y*+1, *z*−1; (vi) −*x*+2, −*y*+1, −*z*.
